# ABSP: an automated R tool to efficiently analyze region-specific CpG methylation from bisulfite sequencing PCR

**DOI:** 10.1093/bioinformatics/btad008

**Published:** 2023-01-11

**Authors:** Marie Denoulet, Mathilde Brulé, François Anquez, Audrey Vincent, Julie Schnipper, Eric Adriaenssens, Robert-Alain Toillon, Xuefen Le Bourhis, Chann Lagadec

**Affiliations:** CNRS, Inserm, CHU Lille, UMR9020-U1277 - CANTHER—Cancer Heterogeneity Plasticity and Resistance to Therapies, University of Lille, Lille F-59000, France; Institut pour la Recherche sur le Cancer de Lille (IRCL), Lille F-59000, France; CNRS, Inserm, CHU Lille, UMR9020-U1277 - CANTHER—Cancer Heterogeneity Plasticity and Resistance to Therapies, University of Lille, Lille F-59000, France; Institut pour la Recherche sur le Cancer de Lille (IRCL), Lille F-59000, France; CNRS, UMR 8523 - PhLAM—Physique des Lasers Atomes et Molécules, University of Lille, Lille F-59000, France; CNRS, Inserm, CHU Lille, UMR9020-U1277 - CANTHER—Cancer Heterogeneity Plasticity and Resistance to Therapies, University of Lille, Lille F-59000, France; Laboratory of Cellular and Molecular Physiology, UR UPJV 4667, University of Picardie Jules Verne, Amiens 80000, France; CNRS, Inserm, CHU Lille, UMR9020-U1277 - CANTHER—Cancer Heterogeneity Plasticity and Resistance to Therapies, University of Lille, Lille F-59000, France; CNRS, Inserm, CHU Lille, UMR9020-U1277 - CANTHER—Cancer Heterogeneity Plasticity and Resistance to Therapies, University of Lille, Lille F-59000, France; CNRS, Inserm, CHU Lille, UMR9020-U1277 - CANTHER—Cancer Heterogeneity Plasticity and Resistance to Therapies, University of Lille, Lille F-59000, France; CNRS, Inserm, CHU Lille, UMR9020-U1277 - CANTHER—Cancer Heterogeneity Plasticity and Resistance to Therapies, University of Lille, Lille F-59000, France; Institut pour la Recherche sur le Cancer de Lille (IRCL), Lille F-59000, France

## Abstract

**Motivation:**

Nowadays, epigenetic gene regulations are studied in each part of the biology, from embryonic development to diseases such as cancers and neurodegenerative disorders. Currently, to quantify and compare CpG methylation levels of a specific region of interest, the most accessible technique is the bisulfite sequencing PCR (BSP). However, no existing user-friendly tool is able to analyze data from all approaches of BSP. Therefore, the most convenient way to process results from the direct sequencing of PCR products (direct-BSP) is to manually analyze the chromatogram traces, which is a repetitive and prone to error task.

**Results:**

Here, we implement a new R-based tool, called ABSP for analysis of bisulfite sequencing PCR, providing a complete analytic process of both direct-BSP and cloning-BSP data. It uses the raw sequencing trace files (*.ab1*) as input to compute and compare CpG methylation percentages. It is fully automated and includes a user-friendly interface as a built-in R shiny app, quality control steps and generates publication-ready graphics.

**Availability and implementation:**

The ABSP tool and associated data are available on GitHub at https://github.com/ABSP-methylation-tool/ABSP.

**Supplementary information:**

Supplementary data are available at *Bioinformatics* online.

## 1 Introduction

Aside from transcription factor regulations, gene expression can also be activated or repressed by epigenetic modifications directly on nucleotides (DNA methylation) or histones (methylation, acetylation…). In vertebrates, epigenetic regulation is essential to regulate genomic imprinting, X chromosome inactivation, development regulation, cell differentiation and genome integrity preservation. DNA methylation can affect cytosine and adenine but mostly occurs on a cytosine followed by a guanine (CpG site). The effect of these modifications on gene transcription has been observed when several grouped CpG within a DNA region, so-called CpG islands, are modified altogether (Greenberg and Bourc'his, 2019; Jones, 2012). Specific enzymes, DNA methyltransferases (DNMT1, DNMT3A and DNMT3B), transfer a methyl group (CH_3_) from S-adenosyl methionine (SAM) on the C5 position of the pyrimidine ring, converting cytosine (C) into 5-methylcytosine (5mC).

Among other methods, the Bisulfite Sequencing PCR (BSP) is the most accessible and conventional method to evaluate methylation levels at single CpG resolution in a mix of DNA molecules (Clark *et al.*, 1994; Frommer *et al.*, 1992). Even if broad methods have been developed to study DNA methylation, the BSP technique has the benefit of a great sensitivity at a very low cost, compared to other methods using next-generation sequencing (NGS) technologies, more sensitive but costly. The BSP assay is thereby the most suited one to quantify DNA methylation of a specific region when large-scale NGS methods are not necessary, especially to get rapid preliminary results or to validate methylation data from screening experiments such as reduced representation bisulfite sequencing at specific loci (Chen *et al.*, 2022; Dehdari *et al.*, 2022; Pajares *et al.*, 2021).

DNA methylation estimation methods using bisulfite conversion are based on the selective deamination of cytosine residues by sodium bisulfite treatment, transforming cytosines into uracils whereas 5mCs are not affected and remain cytosines (Frommer *et al.*, 1992; Hayatsu *et al.*, 1970). Subsequently, the polymerase chain reaction (PCR) regenerates thymines instead of unmethylated cytosines, as both are complementary to adenines, while 5mCs remain cytosines. Therefore, the original methylated cytosines are distinguishable from the unmethylated ones through Sanger sequencing.

**Fig. 1.**
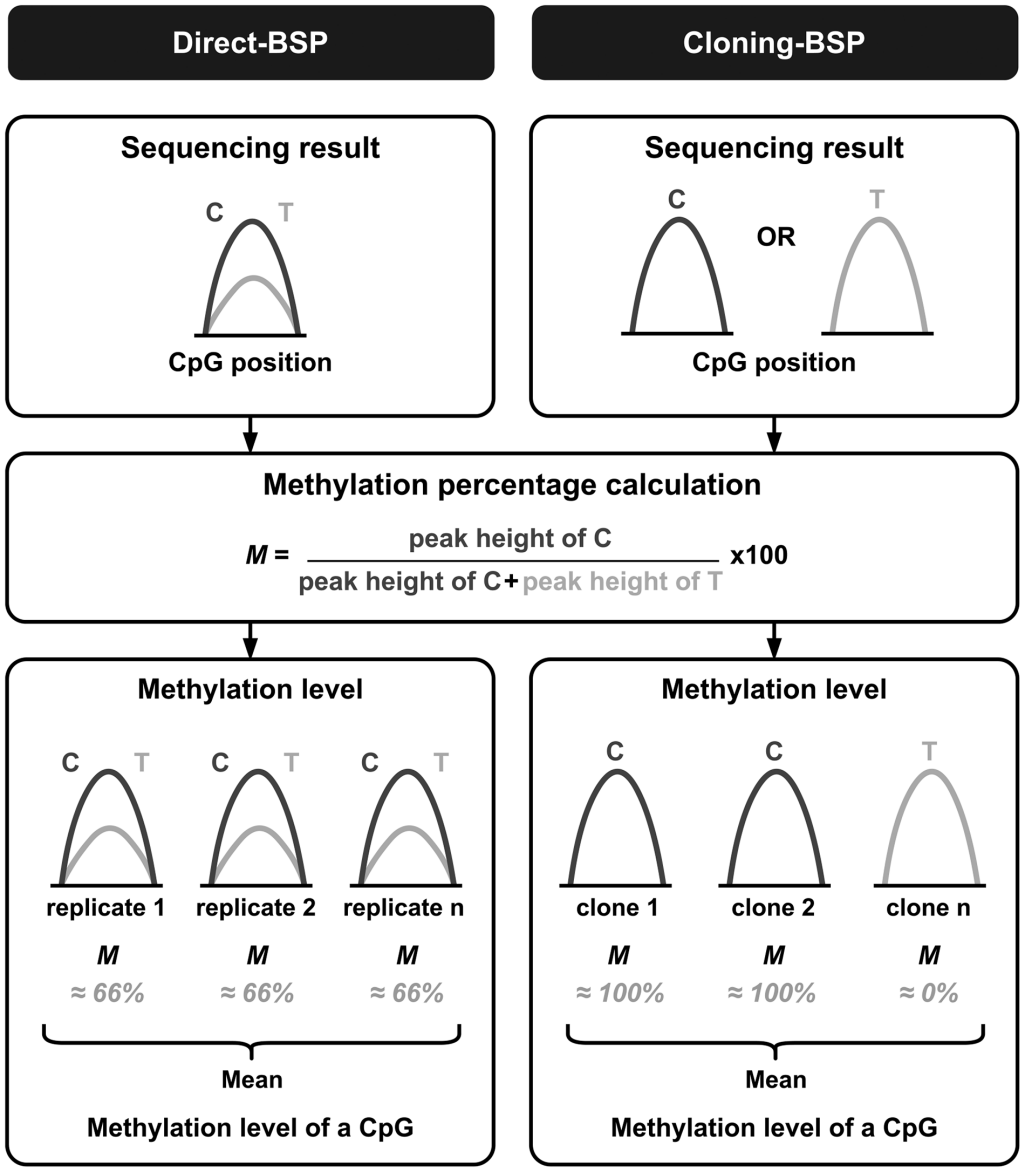
Analysis strategy differences between the two bisulfite sequencing PCR approaches, direct-BSP and cloning-BSP. As the direct-BSP method consists in sequencing the mix of PCR products, the methylation calculation based on chromatogram C/T peak heights gives directly an estimation of the CpG methylation level that can be replicated for statistical significance determination. In the cloning-BSP method, as the CpG methylation status in each clone is revealed by the chromatogram peak heights as well, the sequencing of several clones gives an estimation of the CpG methylation proportion

Two approaches to BSP have been described in the literature: direct BSP and cloning BSP (Chatterjee *et al.*, 2017). The direct-BSP approach consists of sequencing PCR products directly after PCR amplification of bisulfite-converted DNA. As a mix of DNA molecules with different CpG methylation statuses is being sequenced simultaneously, the quantification of CpG methylation can be assessed in the same way as the quantification of a single nucleotide polymorphism (Qiu *et al.*, 2003). Thereby, from the chromatogram trace file, the peak heights ratio of cytosine and thymine signals are used to determine the proportion of methylated cytosines compared to unmethylated ones at CpG sites (Fig. 1) (Jiang *et al.*, 2010; Lewin *et al.*, 2004; Parrish *et al.*, 2012).

In the cloning-BSP approach, PCR products are cloned in vectors and used to transform bacteria. Amplified vectors from individual colonies are extracted for sequencing. Hence, the sequencing of a unique clone, reveals the methylation status of each CpG site of a single PCR product (Li and Tollefsbol, 2011). In the literature, about 10 clones are usually sequenced to get an estimation of the CpG methylation levels of a DNA population with 10–20% accuracy (Chen *et al.*, 2022; Li and Tollefsbol, 2011).

In terms of analysis, tools have been developed to analyze cloning-BSP results, exclusively relying on the base-called sequence from the sequencing. For instance, MethTools 2.0, BiQ Analyzer, QUMA (quantification tool for methylation analysis) and BISMA (bisulfite sequencing DNA methylation analysis) can be cited (Bock *et al.*, 2005; Grunau *et al.*, 2000; Kumaki *et al.*, 2008; Rohde *et al.*, 2010). These tools have been designed to process cloning-BSP data and cannot analyze direct-BSP results as they were not conceived to use the four-dye signal intensity values from chromatograms as an input to interpret the results. Indeed, they determine the methylation statuses of CpG sites of each clone and then calculate the ratio between methylated and unmethylated clones to estimate CpG methylation proportions in the biological sample.

The cloning-BSP approach is mostly used since the direct-BSP one is generally considered less quantitative, due to differences in labeled terminator nucleotides (ddNTPs) incorporation efficiencies and differences in signal relative intensities between the four dyes (Chhibber and Schroeder, 2008; Mikeska *et al.*, 2010). Yet, studies claim that 10 clones are not sufficient to obtain a statistically significant estimation of DNA methylation levels and prone to the direct sequencing of PCR products (Mühlisch *et al.*, 2007; Paul and Clark, 1996; Voss *et al.*, 1998). Besides, direct-BSP is efficient and avoids the multiplication of subclones sequencing costs; it is therefore particularly useful for methylation quantification studies with many samples such as cohorts, or for validation of potential targets identified through screening experiments (Moschny *et al.*, 2020; Schiele *et al.*, 2021).

In the context of The Human Epigenome Project by the Human Epigenome Consortium, the direct-BSP approach was selected to map the CpG methylation levels along the genome for high throughput and cost-effectiveness reasons. Consequently, in 2004, Lewin *et al.* developed an algorithm called ESME (Epigenetic Sequencing Methylation analysis software), to estimate methylation levels from the four-dye chromatogram trace files. However, the software is not up-to-date with the current BSP technology and suffers from accessibility issues as its installation and operation require qualified expertise in a Linux operating system (Akika *et al.*, 2017). So, nowadays, the most convenient way to analyze direct-BSP data still consists in manually retrieving the peak heights to compute methylation percentages of CpG sites, which is time-consuming (dependent on the number of samples and CpG sites per sample), repetitive, prone to errors and does not include valuable quality control over sequencing data (Jiang *et al.*, 2010; Martisova *et al.*, 2021; Parrish *et al.*, 2012).

Additionally, a step further is required for better visualization and comparison of methylation differences. Once methylation levels are obtained, some graphical visualization of methylation data can be generated, by using a web-based tool called Methylation plotter for example, as well as comparative statistics (Mallona *et al.*, 2014).

Existing tools are not sufficient to provide a full analytic process of BSP results, especially for direct-BSP experiments, in the context of preliminary data or large studies for which the cloning is not appropriate. As it is relevant to sequence the PCR products to estimate methylation percentages before committing to the cloning step, the choice was to apply the same method for both direct-BSP and cloning-BSP results to ensure continuity in the analytic process. By using our new tool ABSP (analysis of bisulfite sequencing PCR), both approaches of BSP can be analyzed to generate methylation visualization plots and comparative statistics, in an automated and controlled manner, from the Applied Biosystems, Inc. Format (ABIF) sequencing files (*.ab1*).

## 2 Approach

For direct-BSP, ratios of the peak heights of the two co-existing C and T signals at CpG positions are used to evaluate the proportion of methylated cytosines (Fig. 1) (Jiang *et al.*, 2010; Lewin *et al.*, 2004; Parrish *et al.*, 2012). The same method can be applied to analyze the subclones sequencings: as the ratio of signal peak heights can either be around 0% or 100%, its calculation reveals the CpG methylation status of individual DNA molecules. Therefore, PCR replicates or clone analysis can give statistical meanings of the degree of methylation among the samples (Fig. 1).

**Fig. 2.**
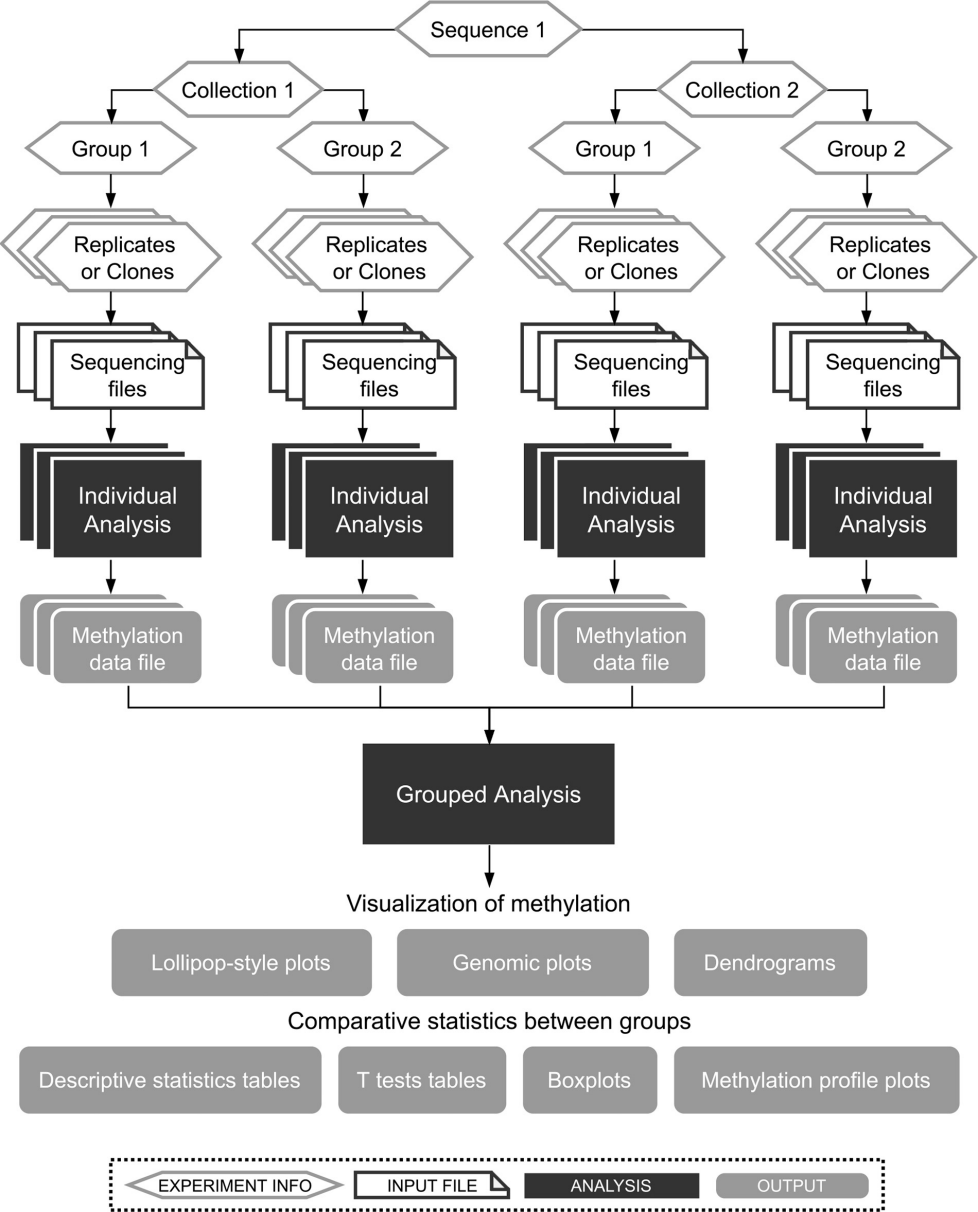
General workflow of ABSP. The analysis is divided into two main steps: the *individual analysis* and *grouped analysis*. The first one serves to control the sequencing quality and compute methylation levels for each individual sample, whereas the second one gathers all sample methylation results to generate visualization plots and process comparative statistics between groups

To fully analyze the BSP experiments, two main steps are required (Fig. 2). First, the CpG methylation levels of each sample have to be estimated using replicates or clones. In our ABSP-developed process, this step is called *individual analysis*. Next, the *grouped analysis* can be run to compare methylation levels between groups and to find methylation differences.

As presented in Figure 2, each sequencing run of a unique PCR product or a unique clone vector is defined by a combination of information used to track, group and compare the sample methylation data: (i) the sequence amplified by PCR (unique primer pair used for the BSP experiment), (ii) the collection, which describes a separation of samples above groups, it means that samples from different collections cannot be compared (e.g. cell lines or organs), (iii) the group, which is the experimental condition to compare (e.g. treatment a or b) and (iv) the replicate number for direct-BSP (repetition identifier) or the clone number for cloning-BSP (clone identifier).

Additionally, sequencing reads from both directions can be provided for each unique DNA sample, using a forward and reverse primer, to maximize the sequence coverage and increase data robustness as both sequencing reads can overlap.

## 3 Materials and methods

### 3.1 General structure

Each one of the two main parts of ABSP, the *individual* and *grouped* analyses, corresponds to an R markdown script (using the *markdown* R package), thereby generating two different types of analysis reports, one specific for individual sample results and the other for grouped samples analysis results. These two analyses can be launched through a shiny app, in which the *individual analysis* tab and the *grouped analysis* tab serve to enter the input parameters, required for report rendering (using the *knitr* R package). Once the inputs are filled and the analysis is launched, the corresponding script processes the analysis, exports several output files and produces the analysis report as an HTML file (*.html* extension), summarizing all the results and serving as a record of them. An additional tab called *multiple analyses* serves to launch several analyses, *individual* ones, *grouped* ones or both, in one click, using filled tables (*.xlsx* or *.csv* files) as input entries.

### 3.2 Individual analysis

The *individual analysis* aims to compute the CpG methylation percentages from the chromatogram trace files of each individual sample at each CpG site, using the signal peak height values.

#### 3.2.1 Input required

**Fig. 3.**
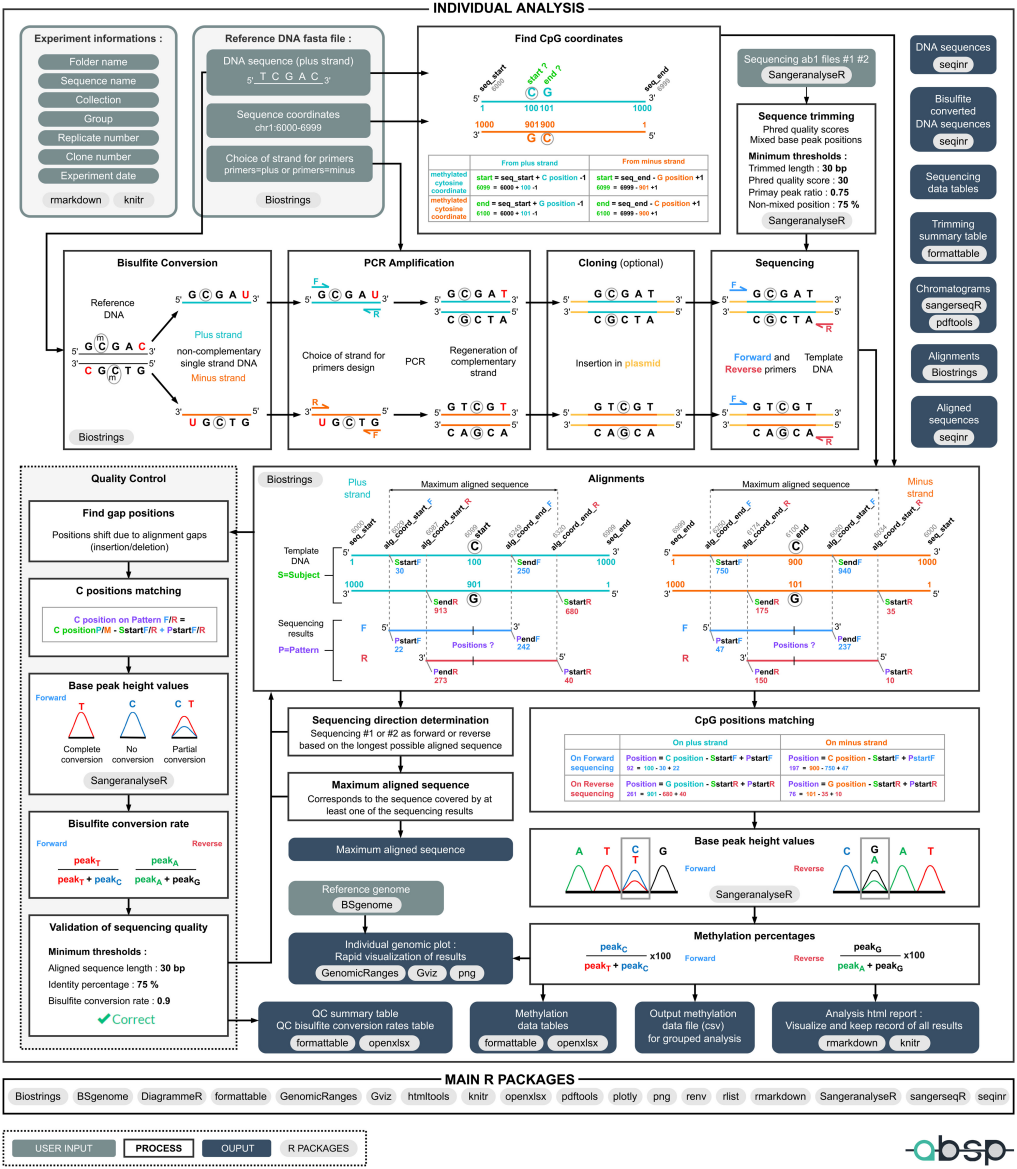
Detailed workflow of the *individual analysis* step. To illustrate the process, an arbitrary reference sequence of 1000 bp was chosen, with genomic coordinates between 6000 and 6999 and a CpG site at positions 100–101 on the plus strand. Other numbers, such as aligned sequences borders, were also arbitrarily chosen for example purposes

Three inputs are required to proceed through the analysis: (i) the sample combination of information, to affiliate the methylation results to the correct sample (Fig. 2), (ii) the genomic reference sequence, its genomic coordinates and the strand amplified during PCR (as the bisulfite converts cytosines into uracils, the two DNA strands are no longer complementary, only one can be amplified with a unique set of primers), which have to be provided in a FASTA file (*.fasta* extension) and (iii) the chromatogram trace files in ABIF format (*.ab1* extension) of the sequencing reads in forward and reverse directions. In Figure 3, the sequencing results are numbered #1 and #2, as the direction must not be specified and will be automatically determined during the analysis.

#### 3.2.2 Processing the reference DNA

First, the CpG positions are retrieved from the reference DNA, and their coordinates are found by correlating positions and reference coordinates (Fig. 3, *Find CpG coordinates* frame). Matches for CG dinucleotides in both plus and minus strands give the start and end positions of each CpG. CpG coordinates are calculated using the start and end coordinates of the reference sequence and CpG positions on the reference sequence (e.g. CpG site at positions 100–101 on plus strand: cytosine coordinate = seq_start + position − 1 = 6000 + 100 − 1 = 6099).

In Figure 3, the second line of process panels represents BSP experiment steps reproduced *in silico* during the analysis. The theoretical bisulfite conversion of the reference DNA is realized using the amplified strand sequence (Fig. 3, *Bisulfite Conversion* frame). As the PCR regenerates the opposite strand of the DNA template, both sequences are retrieved (Fig. 3, *PCR Amplification* frame): (i) sequence from the amplified strand, or sense strand as it will serve as the template for the sequencing in the forward direction (upper strand in Fig. 3) and (ii) the sequence from the opposite strand, or antisense strand as it will serve as the template for the sequencing in the reverse direction (lower strand in Fig. 3) (Fig. 3, *Sequencing* frame).

#### 3.2.3 Trimming of sequencing results

As the extremities of the sequencing reads are prone to off-scale signals and errors, these inaccurate parts must be removed. To determine the correct positions where the sequencing read should be trimmed, two parameters are used: the base calling error probability and the signal peak height (Fig. 3, *Sequencing trimming* frame).

The first trimming method is based on the base calling Phred quality scores, to remove parts susceptible to having base calling errors. This step is performed by the *sangeranalyseR* R package, using the modified Mott’s trimming algorithm (M1 method) with a base calling error probability (*P*) default cutoff of 0.001%, equivalent to a Phred quality score (*Q*) of 30 ($Q=-10\times {\;\text{log}\;}_{10}(P)$) (Chao *et al.*, 2021).

Based on the peak height values from the chromatogram, the second way of trimming aims to remove extremities where signals are mixed. For each base, the ratio of the primary peak height signal over the total of all signals peak height is calculated using the following formula: if $\mathrm{pea}k_{C}>\{\mathrm{pea}k_{A},\mathrm{pea}k_{T},\mathrm{pea}k_{G}\}$, primary peak ratio = $\mathrm{pea}k_{C}/(\mathrm{pea}k_{A}+\mathrm{pea}k_{T}+\mathrm{pea}k_{G}+\mathrm{pea}k_{C})$. A base position is considered as ‘non-mixed’ if the primary peak ratio is above the threshold, set by default to 0.75. Then, all the possible trimmed sequences are obtained by selecting the longest sequence for which the boundaries are of *n* (*n* from 3 to 15) consecutive non-mixed positions. Among those trimmed sequences, the one with a percentage of non-mixed positions above the threshold (default is 75%) with the minimum of consecutive non-mixed positions at boundaries is kept.

Finally, the overlap between both sequences, from the quality score trimming and the mixed base peak trimming, gives the final trimmed sequence used for the following steps. If one of the trimming methods fails or if the final trimmed sequence parameters are below thresholds (length, average Phred score and percentage of non-mixed positions, Fig. 3, *Sequencing trimming* frame), the sequencing will not be used to compute methylation percentages.

#### 3.2.4 Alignments of trimmed sequencing reads with template DNA sequences

To correlate nucleotide positions on the sequencing reads with CpG positions on the template DNA, the alignment of sequences is performed (local pair-wise alignment). In cloning-BSP experiments, sequencing primers are often chosen on the vector backbone. As PCR products can be inserted in either direction, it is crucial to determine the direction of the sequencing within the analytic process. Trimmed sequences are first aligned with both sense and antisense sequences of the converted template DNA. The longest alignment is considered the correct template (Fig. 3, *Alignments* frame).

Knowing the positions of the first nucleotide on template DNA (Subject, S) and sequencing read (Pattern, P), respectively *SstartF*/*PstartF* for forward sequencing and *SstartR*/*FstartR* for the reverse sequencing, a direct correlation is used to find cytosine positions on the trimmed sequencing results. So, as an example, on the forward strand, if the cytosine is at the position 100 on the template (S), the *SstartF *=* *30 and the *PstartF *=* *22, cytosine position = cytosine position on template −*SstartF* + *PstartF *=* *100 − 30 + 22 = 92.

The maximum aligned sequence corresponds to the sequence covered by at least one of the sequencing reads, and its coordinates are determined by the correlation of genomic coordinates and aligned positions (*alg_coord_start* and *alg_coord_end*).

#### 3.2.5 Quality control of the aligned sequencing results

To check the concordance between the template DNA and the sequencing results, the aligned sequences are controlled through several steps: (i) gap positions determination, (ii) C positions matching for bisulfite conversion rate calculation, (iii) retrieval of peak height values for each C position outside CpG sites, (iv) bisulfite conversion rate calculation and (v) validation of the sequencing quality.

**Fig. 4.**
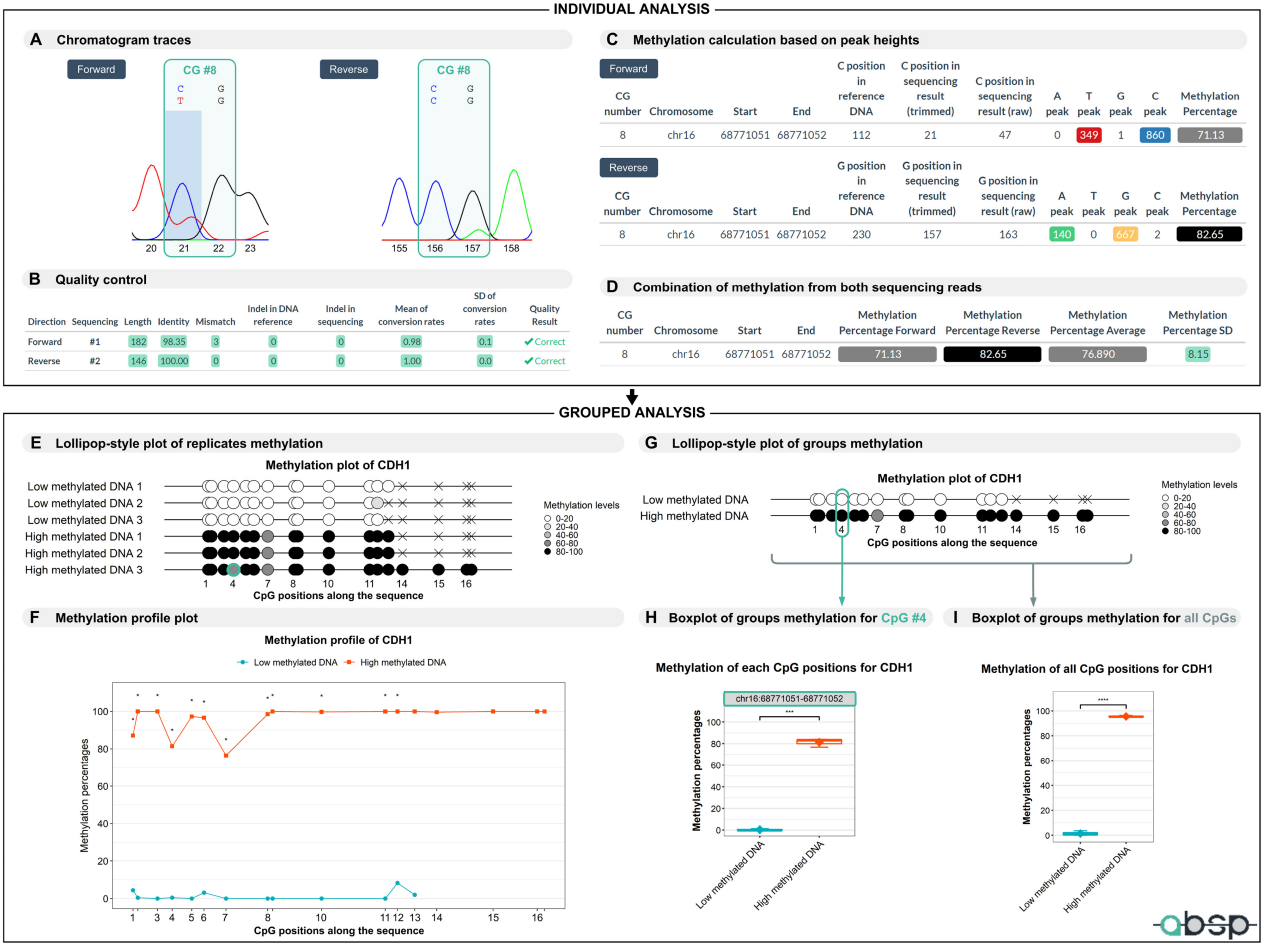
Output results from ABSP analysis. (**A**) Portions of the trimmed chromatogram traces from forward and reverse sequencing reads of the CDH1 sequence in the high-methylated DNA sample, replicate #3. The CG #8 is highlighted and corresponds to the eighth CG dinucleotide of the reference sequence. (**B**) Table of quality control summary after alignments of trimmed sequencing results with the reference sequence. (**C**) Tables of the methylation calculation per CpG using peak height values from both chromatogram traces. (**D**) Table of combined methylation data from both sequencing results, with the methylation average and standard deviation per CpG. (**E**) Lollipop-style plot of CpG methylation levels on the CDH1 sequence, with all the samples displayed and CpG sites placed proportionally to their coordinates. The CG #8 of the high-methylated DNA #3 sample, detailed in the *individual analysis* panel, is highlighted by encircling targeted CG in the lollipop-style plot and corresponds to the fourth CpG site on the covered sequence. The crosses represent unavailable data for CpG sites not covered by the trimmed sequencing results in some samples. (**F**) Methylation profile plot displaying average methylation levels of CpG along the CDH1 sequence in each group as a line plot. Symbols represent significance levels of Kruskal–Wallis test *P*-values (*P ≤ 0.05, **P ≤ 0.01, ***P ≤ 0.001, ****P ≤ 0.0001). (**G**) Lollipop-style plot of average CpG methylation levels on the CDH1 sequence in each group. (**H**) Boxplot of the CpG #4 average methylation levels in each group. (**I**) Boxplot of the methylation percentage means of all CpG positions on the CDH1 sequence in each group. In boxplots, symbols represent significance levels of Student’s *t*-test *P*-values (*P ≤ 0.05, **P ≤ 0.01, ***P ≤ 0.001, **** *P* ≤ 0.0001)

As the retrieval of peak height values for methylation calculation is based on the start positions of aligned sequences, the presence of a gap, insertion or deletion in either the template DNA or the sequencing result, causes a position shift that needs to be corrected for the CpG position matching step. The most important criteria to validate the quality of a sequencing result are the bisulfite conversion efficacy. To assess its efficacy, the bisulfite conversion rate is computed for each cytosine position outside CpG sites and the average rate on the sequence must be higher than the provided threshold (default is 0.9). First, C positions have to be retrieved based on the alignments, with the same method as explained above, by matching the positions of aligned sequences. Then, the peak height values of each base at these positions are used to calculate the bisulfite conversion rates with the following formula, for the forward sequence: bisulfite conversion rate = $\mathrm{pea}k_{T}/(\mathrm{pea}k_{C}+\mathrm{pea}k_{T})$ and for the reverse sequence: bisulfite conversion rate = $\mathrm{pea}k_{A}/(\mathrm{pea}k_{G}+\mathrm{pea}k_{A})$. Finally, the alignments and quality control steps provide the aligned sequence length, identity percentage, mismatches positions, insertion/deletion positions and the average bisulfite conversion rate (Fig. 4B). For a sequencing result to be considered as correct, the length, identity percentage and average bisulfite conversion rate have to be higher than the defined thresholds, set to 30 bp, 75% and 0.9, respectively by default.

#### 3.2.6 Methylation calculation

The methylation percentage of each CpG site is calculated using the peak height values corresponding to the intensity of the dye signal, with the following formula: methylation percentage = $\mathrm{pea}k_{C}/(\mathrm{pea}k_{C}+\mathrm{pea}k_{T})\times 100$ or = $\mathrm{pea}k_{G}/(\mathrm{pea}k_{G}+\mathrm{pea}k_{A})\times 100$ for the forward and reverse sequencing results, respectively (Fig. 4C and D).

#### 3.2.7 Outputs

The main output result of the *individual analysis* is the methylation data table, used as input for the *grouped analysis* afterward. To visualize the methylation levels of the analyzed sample, a plot displaying the genomic sequence, the CpG positions, and the methylation levels as a grey gradient is produced (Supplementary Fig. S1). This genomic plot can serve as a control of the coordinates, as CpG site coordinates must match the sequence colors of CG dinucleotides.

### 3.3 Grouped analysis

#### 3.3.1 Methylation data from individual analysis

As input, the methylation data files saved by the previous *individual analyses* are automatically retrieved based on the selected folder and sequence name. Methylation data from all samples are processed and gathered.

For each individual clone, the methylation percentages found based on signal peak ratios are converted into methylation statuses. By default, for each CpG, a methylation level between 0% and 20% is considered as an unmethylated status and a methylation level between 80% and 100% as a methylated one. Partial methylation, between 20% and 80%, is considered defective and is annotated as not available. For one clone, if the number of partially methylated CpGs is important (above 20% by default) the clone is considered as a potential mix of clones and therefore all of its methylation data is annotated as not available.

#### 3.3.2 Generation of plots to visualize methylation levels

To generate visualization plots, several plot parameters are required: (i) the label type for CpG positions (CpG coordinates, CpG numbers or none), (ii) the collection separation, whether or not samples from different collections have to be displayed on the same plot, (iii) the group order for display and (iv) the sample ordering on the ordinate axis (as it is, by groups, by methylation levels or by clusters).

To represent CpG methylation levels, the lollipop-style plots are largely used in the literature. They illustrate CpG levels as circles with methylation levels either as a black and white scale for clone methylation status or as a grey gradient for methylation level (Fig. 4E and G). Most plots generated by ABSP were built using the functions of the Methylation plotter tool as a reference (Mallona *et al.*, 2014).

As for the *individual analysis*, methylation levels are also pictured by genomic plots, displaying the genomic sequence, CpG positions and CpG methylation of samples as a grey-scale heatmap along the sequence (Supplementary Fig. S2).

#### 3.3.3 Comparative statistics

As the purpose of the BSP experiment is to compare results from different conditions over methylation levels, several outputs are generated: tables with the two-by-two comparisons of groups with Student’s *t*-test *P*-values, boxplots representing the methylation means of each CpG, and boxplots with the means of all CpG analyzed gathered with Student’s *t*-test *P*-values as well (Fig. 4H and I, Supplementary Fig. S3), and finally methylation profile plots displaying the methylation levels as line plots along the sequence with Kruskal–Wallis *P*-values per CpG, to identify the sites with significant differences among the groups (Fig. 4F) (Mallona *et al.*, 2014).

## 4 Application

Both high-methylated and low-methylated human genomic DNA (80-8061-HGHM5 and 80-8062-HGUM5 from EpigenDx) were treated with sodium bisulfite and cleaned up. An upstream promoter region of the CDH1 gene, covering 17 CpG sites was amplified through a touchdown PCR protocol using specific primers, 5′ tailed with standard primers T3 or BGH Reverse. The 259 bp long amplicons were directly sequenced in both directions, in triplicates to allow statistical analysis (for additional details on the method, see the Supplementary Materials).

Sequencing results were processed and analyzed using the ABSP workflow described for direct-BSP analysis. Essential results from the *individual analysis* and *grouped analysis* reports are respectively displayed in Figure 4 top and bottom panels.

The CG #8 from the high-methylated DNA #3 sample is displayed to illustrate the analysis process and outputs (Fig. 4). After alignment with the reference sequence and validation of both sequencing results through quality control (Fig. 4B), the peak height values corresponding to each base at the CG #8 cytosines positions are retrieved (Fig. 4C). The C and T peak heights are used to compute the methylation percentage from the forward sequencing, and the A and G peak heights from the reverse sequencing, as displayed in Figure 4C. After combining methylation results from both sequencing reads, the average methylation percentage and standard deviation are computed and these data will be used in the *grouped analysis* (Fig. 4D). For the CG #8 illustrated in Figure 4, the sequencing analysis reveals methylation of 71.13% for the forward result and 82.65% for the reverse, given an average methylation of 76.89% (Fig. 4C and D). As CG position numbers are determined based on the reference DNA in the *individual analysis* and are then reset in the *grouped analysis*, the previously described CG #8 corresponds now to the CpG site #4 covered by at least one of the sequencing.

As the *grouped analysis* aims to facilitate the interpretation of methylation data from all samples, several graphics are generated. First, in the lollipop-style plot displaying methylation of all samples, the difference between the low-methylated and high-methylated samples is clearly visible thanks to the grey scale (Fig. 4E). In addition, missing points, inconsistent methylation levels between replicates or clones, and methylation patterns can be easily found on this type of plot. In the high-methylated DNA #3 sample, it is particularly noticeable that the CpG site #4 has a slightly lower methylation level (76.89%) compared to the two other high-methylated DNA replicates (Fig. 4E, green circle). For unknown reasons, the forward sequencing reads were not clean enough and failed to pass the trimming and/or quality control steps for 5 out of 6 samples, explaining the missing data points, covered neither by the forward sequencing read nor by the beginning of the reverse sequencing read. For a robust comparison of methylation between groups, the methylation profile plot indicates the CpG sites for which the difference in methylation level is significant among groups, which is the case here for all the CpG covered in the two groups (Fig. 4F). Additionally, the lollipop-style plot displaying the methylation means of groups provides less information but gives an efficient overview of methylation differences between groups (Fig. 4G).

To complement the comparative analysis, boxplots of each CpG site and the boxplot of means of CpG methylation, indicate the distribution of methylation among the groups as well as the significance of methylation differences between groups two-by-two (Fig. 4H and I). The CpG site #4 has a methylation percentage of 0.47% (±0.81%) in low-methylated DNA and 81.44% (±3.96%) in high-methylated DNA, with a statistical *P*-value of 0.00051. Also, the mean methylation rate of the sequence CpG is 1.43% (±1.87%) in low-methylated DNA and 95.54% (±0.67%) in high-methylated DNA, with a statistical *P*-value of 2.34e^−05^, confirming the difference of methylation of the analyzed sequences. All the data associated with this example (inputs, reports and outputs) are provided along with the ABSP files, available at https://github.com/ABSP-methylation-tool/ABSP.

## 5 Discussion

For this work, we developed a modern and useful tool to analyze both the direct and cloning approaches of BSP. As ESME software is the reference for such studies, we compared results obtained from ESME to ABSP and found several differences. First, ESME performs a normalization of cytosines signals as it assumes that the less frequent base signals are overscaled by the basecaller (example of ESME results in Supplementary Fig. S4) (Lewin *et al.*, 2004). However, ESME was developed in the early stages of the BSP technology, and nowadays basecallers have been improved and do not exaggerate the missing base, its normalization step is therefore no longer required and may introduce biases in methylation percentages calculated (Methylation Analysis by Bisulfite Sequencing: Chemistry, Products and Protocols from Applied Biosystems, 2007, https://assets.thermofisher.com/TFS-Assets/LSG/manuals/cms_039258.pdf). Hence, ABSP does not apply any changes to the peak height values retrieved from chromatogram trace data, as performed in other studies (Jiang *et al.*, 2010; Parrish *et al.*, 2012) (comparison of ESME and ABSP results in Supplementary Table S1).

Also, ABSP provides several key advantages compared to ESME. The main added values of ABSP is a built-in comparative analysis step, including methylation data visualization, with ready-to-publish graphics and statistical tests, to help researchers answer the experimental hypothesis. Moreover, by being able to process both direct-BSP and cloning-BSP data, ABSP provides an analysis continuity, from preliminary data by direct-BSP up to validation by cloning-BSP. In terms of accessibility, as only R and RStudio are required, ABSP can operate on every operating system supporting both software (Windows, Linux and macOS). More importantly, the full automation of the analysis and the user-friendly interface makes ABSP accessible to users without expertise in R, such as most of the biologists.

Yet, ABSP is still an adaptable tool for R accustomed users, as the code can be modified to be adapted to experiments or user needs. For example, minor modifications such as threshold adjustments or plot customization are possible (help to implement the modifications is included in the user guide). Other major modifications such as expanding the analysis to include CHG and CHH sites (H being non-Gs bases), can be developed and implemented to ABSP code in a future version, to fit it to DNA methylation study in plant models. Altogether, ABSP provides a new and easy way to process sequencing data from BSP experiments and help researchers to compare methylation profile for a given sequence.

## Supplementary Material

btad008_Supplementary_Data
