# Supplementary material for: ABSP: an automated R tool to efficiently analyze region-specific CpG methylation from bisulfite sequencing PCR
Source: Bioinformatics. 2023 Jan 11;39(1):btad008. doi: 10.1093/bioinformatics/btad008 (PMC9846423; doi:10.1093/bioinformatics/btad008)
Supplement: btad008_Supplementary_Data [file btad008_supplementary_data.pdf]

Supplementary Materials

ABSP: an automated R tool to efficiently analyze region-specific CpG methylation from bisulfite sequencing PCR.

Marie Denoulet [1,2], Mathilde Brulé [1,2], François Anquez [3], Audrey Vincent [1], Julie Schnipper [4], Eric Adriaenssens [1], Robert-Alain Toillon [1], Xuefen Le Bourhis [1] and Chann Lagadec [1,2]

[1] Univ. Lille, CNRS, Inserm, CHU Lille, UMR9020-U1277 - CANTHER - Cancer Heterogeneity Plasticity and Resistance to Therapies, F-59000 Lille, France.
[2] Institut pour la Recherche sur le Cancer de Lille (IRCL), 59000 Lille, France.
[3] CNRS, UMR 8523 - PhLAM - Physique des Lasers Atomes et Molécules, University of Lille, 59000 Lille, France.
[4] Laboratory of Cellular and Molecular Physiology, UR UPJV 4667, University of Picardie Jules Verne, Amiens, France.

1 Supplementary Information

Two DNA samples, high-methylated and low-methylated human genomic DNA (80-8061-HGHM5 and 80-8062-HGUM5 from EpigenDx), were treated with sodium bisulfite. 1.4 µg of each DNA sample was mixed with 0.3 M of NaOH and incubated at 50°C for 20 min. Then, DNA solutions were treated with a 2.5 M of sodium bisulfite / 125 mM of hydroquinone pH 5.0 solution, at 70°C for 3 h.

The single-stranded bisulfite converted DNA was then cleaned up using the NucleoSpin Gel and PCR Clean-up kit (Macherey-Nagel) following the manufacturer's instructions and converted DNA samples were stored at -80°C before PCR amplification.

An upstream promoter region of the CDH1 gene was amplified using a touchdown PCR protocol and directly sequenced in both directions.

Each PCR reaction was performed with 1.5 µL of RDA buffer (670 mM of Tris pH 8.8, 160 mM of $(NH_4)_2SO_4$, 100 mM of β-Mercaptoethanol, and 1 mg/mL of Bovine Serum Albumin from NEB), 1.2 µL of 500 mM $MgCl_2$, 0.075 µL of 10 mM dNTP, 0.1 µL of 5 U/µL Taq DNA polymerase recombinant (Invitrogen), 0.5 µL of each primer at 5 µM, 1 µL of bisulfite converted DNA sample and 10.125 µL of water.

The touchdown PCR protocol was composed of 50 cycles of: 20 s at 95°C, 30 s at annealing temperature, and 2 min at 72°C. The annealing temperature varies from 60°C for 10 cycles, to 59°C, 58°C, 57°C, and 56°C for 1 cycle each, and 55°C for 36 cycles.

The specific primer sequences are tailed at 5'end with a standard primer, T3 or BGH Reverse (underlined), forward primer: 5'-<u>AATTAACCCTCACTAAAGGG</u>TTTAGTAATTTTAGGTTA-GAGGGTTAT-3' and reverse primer: 5'-<u>TAGAAGGCACAGTCGAGG</u>AAACTCACAAA-TACTTTACAATTCC-3'. The amplicons were first validated by gel electrophoresis migration and then directly sequenced using the T3 (5'-<u>AATTAACCCTCACTAAAGGG</u>-3') and BGH Reverse (5'-<u>TAGAAGGCACAGTCGAGG</u>-3') primers, for forward and reverse directions, respectively. Triplicates of sequencing runs have been performed to allow statistical analysis.

The CDH1 amplified region is located on the plus strand at coordinates chr16:68771007-68771227 (reference human genome hg19) (221 bp) and covers 17 CpG sites. With the addition of the primers 5'tails T3 (20 bp) and BGH Reverse (18 bp), the length of the amplicon is 259 bp.

Amplicon sequence (bisulfite converted sequence with CpG sites considered as methylated

highlighted in grey and standard primers T3 and BGH Reverse underlined):
5’-AATTAACCCTCACTAAAGGGTTTAGTAATTTTAGGTTAGAGGGTTATCGCGTTTATG
CGAGGTCGGGTGGGCGGGTCGTTAGTTTCGTTTTGGGGAGGGGTTCGCGTTGTTGATTG
GTTGTGGTCGGTAGGTGAATTTTTAGTTAATTAGCGGTACGGGGGGCGGTGTTTTCGGG
GTTTATTTGGTTGTAGTTACGTATTTTTTTTTAGTGGCGTCGGAATTGTAAAGTATTTGT
GAGTTTCCTCGACTGTGCCTTCTA-3’

The ABSP analysis of results was performed using as a reference DNA sequence the plus strand genomic sequence at coordinates chr16:68770940-68771280 (reference human genome hg19):
5’-CCACCGGCGGGGCTGGGATTCGAACCCAGTGGAATCAGAACCGTGCAGGTCCCATA
ACCCACCTAGACCCTAGCAACTCCAGGCTAGAGGGTCACCGCGTCTATGCGAGGCCGG
GTGGGCGGGCCGTCAGCTCCGCCCTGGGGAGGGGTCCGCGCTGCTGATTGGCTGTGGC
CGGCAGGTGAACCCTCAGCCAATCAGCGGTACGGGGGGCGGTGCCTCCGGGGCTCACC
TGGCTGCAGCCACGCACCCCCTCTCAGTGGCGTCGGAACTGCAAAGCACCTGTGAGCT
TGCGGAAGTCAGTTCAGACTCCAGCCCGCTCCAGCCCGGCCCGACCCGACCGC-3’

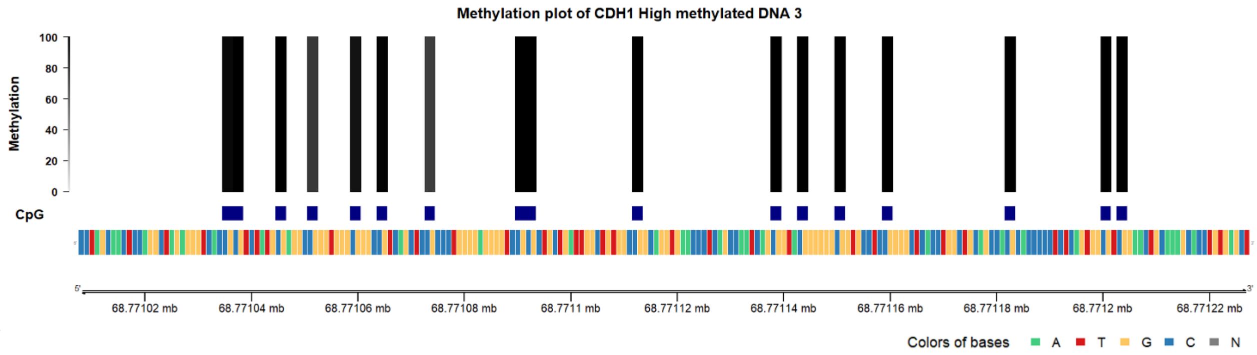

2 Supplementary Figures

Fig. S1 Genomic plot of CDH1 methylation levels in the high-methylated DNA 3 sample from the individual analysis. Methylation percentages are displayed as a linear grey gradient along the genomic sequence.

Fig. S2 Genomic plot of CDH1 methylation levels in all the analyzed samples from the grouped analysis. Methylation percentages are displayed as a linear grey gradient along the genomic sequence. A methylation of 0% is displayed as a light grey color while missing data points are displayed as a white color. (**A**) The methylation percentages from each

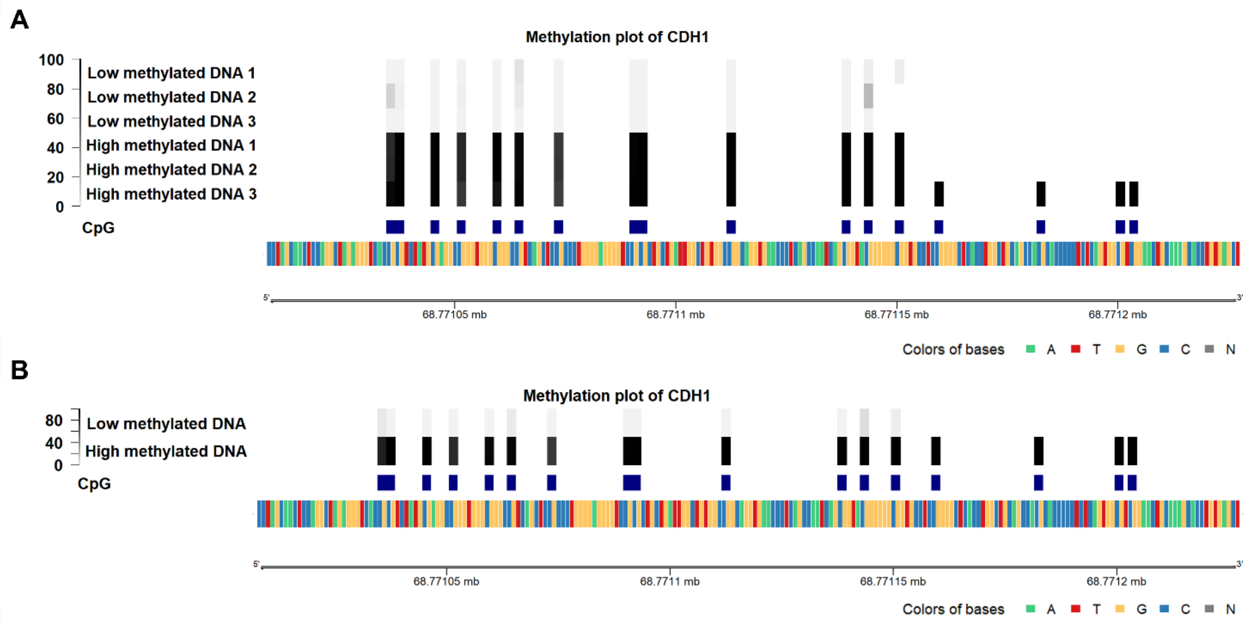

individual sample are represented on the plot. (**B**) The means of methylation of each group per CpG sites are represented on the plot.

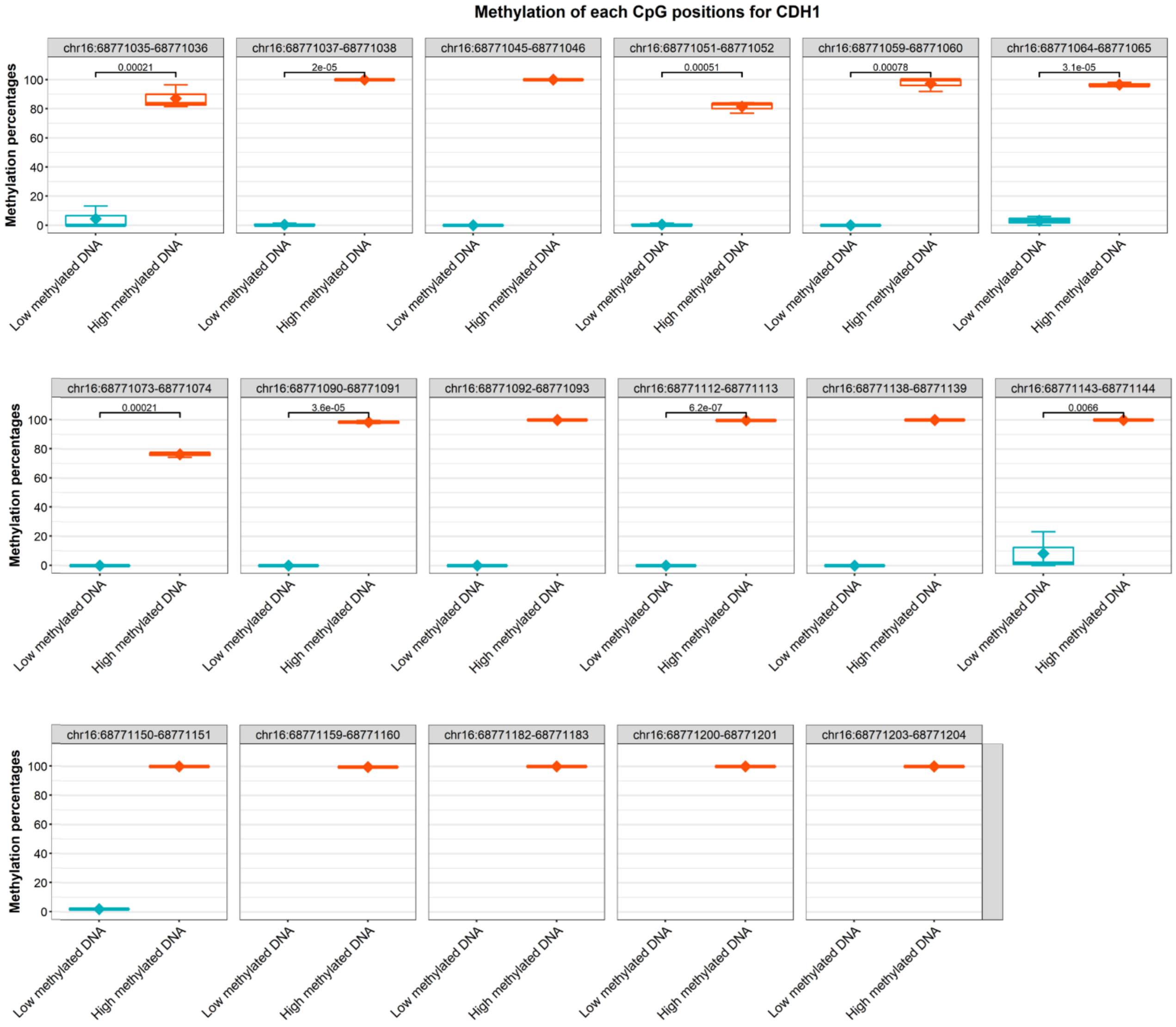

Fig. S3 Boxplots of CDH1 methylation distribution between groups for each CpG position. Some data points are missing in the low-methylated DNA samples for the last 4 CpG sites.The Student's T-test *p*-values are displayed at CpG sites for which the T-test could be performed on the data. The original figure was rearranged to fit to page.

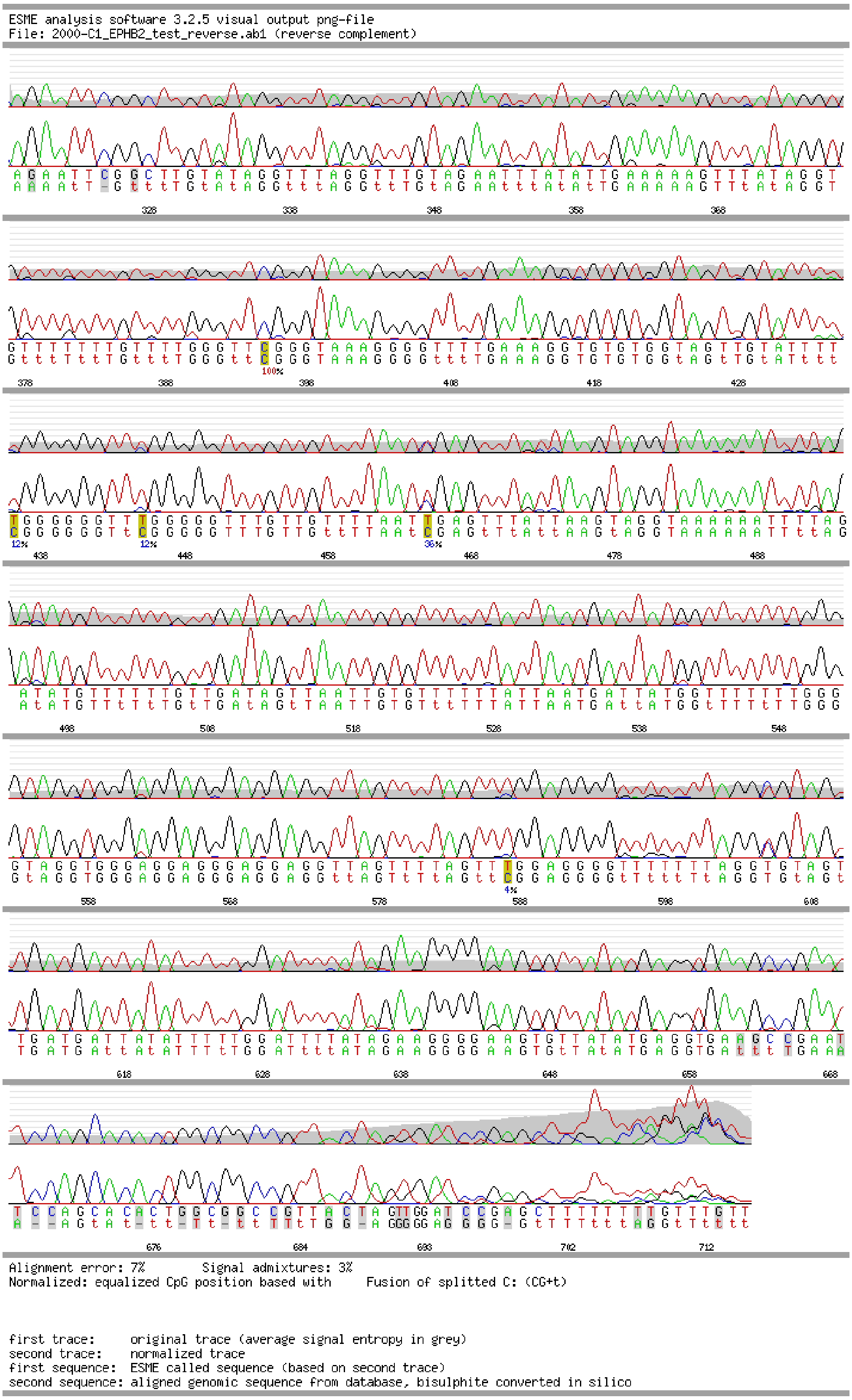

Fig. S4 Example of a trace data file resulting from an ESME analysis. A direct-BSP experiment was conducted on a region in the EPHB2 gene, located at coordinates chr1:23176079-23176408 (hg19). The sequencing run from the reverse primer was analyzed by the ESME software. The file name contains the “C1” mention to indicate the use of a C-rich (reverse) primer, designed from the plus/top strand (1) of genomic DNA. Hence the trace data are representing the reverse complement of the original sequencing result. The first chromatogram trace corresponds to the orignal trace without normalization and the second to the normalized trace. CpG sites are higlighted in yellow and methylation percentages calculated from traces are indicated below each one of them.

CpG number	CpG coordinates	C position on reference sequence chr1:23176000-23176999	Methylation percentage from ESME	Methylation percentage from ABSP
CpG #1	chr1:23176394-23176395	395	100%	100%
CpG #2	chr1:23176435-23176436	436	12%	30%
CpG #3	chr1:23176444-23176445	445	12%	31%
CpG #4	chr1:23176464-23176465	465	36%	48%
CpG #5	chr1:23176586-23176587	587	4%	21%

Tab. S1 Comparison of methylation percentages obtained from both the ESME and ABSP analysis on the 5 CpG sites within the chr1:23176079-23176408 (hg19) region located in the EPHB2 gene.
